# Exogenously Applied Rohitukine Inhibits Photosynthetic Processes, Growth and Induces Antioxidant Defense System in *Arabidopsis thaliana*

**DOI:** 10.3390/antiox11081512

**Published:** 2022-08-03

**Authors:** Sajad Ahmed, Mohd Asgher, Amit Kumar, Sumit G. Gandhi

**Affiliations:** 1 Indian Institute of Integrative Medicine, Canal Road, Jammu 180001, Jammu and Kashmir, India; amitkumar@iiim.ac.in; 2 Department of Botanical and Environmental Sciences, Guru Nanak Dev University, Amritsar 143005, Punjab, India; 3 Plant Physiology and Biochemistry Laboratory, Department of Botany, School of Biosciences and Biotechnology, Baba Ghulam Shah Badshah University, Rajouri 185234, Jammu and Kashmir, India; asghermohd@gmail.com; 4 Academy of Scientific and Innovative Research (AcSIR), Ghaziabad 201002, Uttar Pradesh, India

**Keywords:** antioxidants, *Arabidopsis*, metabolome, ROS, rohitukine

## Abstract

The secondary metabolite rohitukine has been reported in only a few plant species, including *Schumanniophyton magnificum*, *S. problematicum*, *Amoora rohituka*, *Dysoxylum acutangulum* and *D. gotadhora*. It has several biological activities, such as anticancer, anti-inflammatory, antiadipogenic, immunomodulatory, gastroprotective, anti-implantation, antidyslipidemic, anti-arthritic and anti-fertility properties. However, the ecological and physiological roles of rohitukine in parent plants have yet to be explored. Here for the first time, we tried to decipher the physiological effect of rohitukine isolated from *D. gotadhora* on the model system *Arabidopsis thaliana*. Application of 0.25 mM and 0.5 mM rohitukine concentrations moderately affected the growth of *A. thaliana*, whereas a remarkable decrease in growth and the alteration of various morphological, physiological and biochemical mechanisms were observed in plants that received 1.0 mM of rohitukine as compared to the untreated control. *A. thaliana* showed considerable dose-dependent decreases in leaf area, fresh weight and dry weight when sprayed with 0.25 mM, 0.5 mM and 1.0 mM of rohitukine. Rohitukine exposure resulted in the disruption of photosynthesis, photosystem II (PSII) activity and degradation of chlorophyll content in *A. thaliana*. It also triggered oxidative stress in visualized tissues through antioxidant enzyme activity and the expression levels of key genes involved in the antioxidant system, such as superoxide dismutase (SOD), peroxidase (POD) and ascorbate peroxidase (APX). Rohitukine-induced changes in levels of metabolites (amino acids, sugars, organic acids, etc.) were also assessed. In light of these results, we discuss (i) the likely ecological importance of rohitukine in parent plants as well as (ii) the comparison of responses to rohitukine treatment in plants and mammals.

## 1. Introduction

Plants synthesize a large number of small molecules for use in defence against biotic and abiotic stresses [1,2,3]. These secondary phytochemicals have commercial applications in pharmaceuticals, flavourings and fragrances, insecticides, etc. [4,5]. They are divided into three main groups: phenolic compounds, terpenoids and nitrogen-containing alkaloids [6]. Usually, alkaloids contain basic nitrogen, derived from an amino acid or purine/pyrimidine, while in some pseudoalkaloids the source of nitrogen is a transamination reaction [7]. Among alkaloids, the chromone alkaloids are a unique group with many biological activities, structurally consisting of a nitrogen system (pyridine, piperidine, pyrrolidine) linked to the A-ring of chromone [8].

The chromone alkaloid rohitukine was isolated for the first time from *A. rohituka* [9] and its structure has been shown to be that of a highly polar molecule [9,10,11]. It is restricted to only two plant families: Rubiaceae (*Schumanniophyton magnificum* and *S. problematicum*) and Meliaceae (*Dysoxylum gotadhora, D. acutangulum* and *Amoora rohituka*) [12]. Both families are mainly distributed in tropical areas of the world [13]. *D. gotadhora* (Indian white cedar) has been in use in Ayurvedic and other traditional systems of indigenous medicine for many years to treat diabetes, jaundice, leucorrhoea, piles, leprosy, osteomyelitis, etc. [14]. *D. gotadhora* is known as the main source of rohitukine. Within the plant body, rohitukine is found to accumulate in leaves, bark, seeds and fruits [15]. The pharmaceutical potential of rohitukine has been deeply assessed in breast, ovarian and lung cancer cell lines, where it inhibits cyclin-dependent kinase (CDK) CDK2/A and CDK9/T1 complexes by blocking their ATP binding sites [10]. In budding yeast, rohitukine induces ROS generation and apoptosis [16]. P-276-00, IIIM-290 and flavopiridol are semisynthetic derivatives of rohitukine.

The rohitukine-inspired molecule flavopiridol has gained considerable attention in the last two decades for its potent cytotoxic activity against a wide range of cancer cell lines [17] and it has now been approved by the European Medicines Agency for the treatment of chronic lymphocytic leukemia (CLL) [18]. Flavopiridol potently inhibits CDKs 1, 2 and 4, causing cell cycle arrest in G1 and G2 phases in mammalian cells [19]. P-276-00 (Piramal Healthcare Limited, Mumbai, India) is another derivative of rohitukine that has advanced into clinical trials for cancer treatment. P-276-00 selectively inhibits CDK4/D1, CDK1/B and CDK9/T1, and its antiproliferative effect has been observed against a wide range of cancer cell lines [20]. IIIM-290, an orally bioavailable anticancer drug that is already being examined in apre-clinical study, is also derived from rohitukine [21,22]. 

Similar to many other medicinal plants, the presence of rohitukine in *D. gotadhora* may likely exert allelopathic effects on neighbouring plants. It has been observed that plants release chemical compounds into their surrounding environment which influence their growth and also contribute to restricting invasion by exotic plant species [23,24]. These phytotoxic compounds are usually biosynthesized in plants as secondary products and many of them have been explored for their pharmacological activities [25,26]. To address the phytotoxic effects of secondary metabolites, many studies have been published which show that secondary metabolites have an impact mainly through damage to photosynthetic machinery and frequent decomposition of photosynthetic pigments, the decrease in photosynthetic pigments leading to blockage of energy/electron transfer and inhibition of ATP synthesis [27,28,29]. Several alkaloids isolated from the medicinal plant *Ruta graveolens* have also been investigated for their photosynthetic inhibitory activities in recipient plants [30]. Antidesmone is a plant secondary metabolite that causes disruption to photosynthetic machinery [31]. Additionally, the secondary compounds of *Satureja hortensis* affect seed germination, morphology and bleach out chlorophyll content in *Amaranthus retroflexus* and *Chenopodium album* [32]. It is quite evident that the targets of toxic plant secondary metabolites are achieved through perturbations to PSII activity [33,34]. In plants, photosynthetic damage is also linked to increased levels of reactive oxygen species (ROS),which indicates oxidative stress [35,36,37]. Increased ROS levels lead to oxidative damage to cells. Similarly, exposure to toxic secondary metabolites also triggers the ROS pathway in recipient plants [38,39]. To resist oxidative stress, plants, upon exposure to such chemicals, alter the activities of antioxidant enzymes, such as SOD, POD, APX and CAT [27,40,41]. 

The above-mentioned findings indicate that rohitukine as well as its semisynthetic derivatives have been explored for their remarkable biological activities in animal cells, wherein rohitukine has been reported to trigger ROS generation and apoptosis [16,42]. However, the phytotoxic effect of rohitukine on the growth and development of plants has not been explored. Therefore, here we tried to understand the morphological, physiological and biochemical changes in *A. thaliana* (a model system) treated with appropriate rohitukine concentrations isolated from *D. gotadhora*. The main objectives of the study were to understand the interference of rohitukine with the antioxidant system of *A. thaliana* and its impacts on photosynthesis. Furthermore, we sought to gain insights into photosynthetic pigments, the phytotoxicity attributed to ROS generation and changes in levels of metabolites, such as amino acids, sugars and other organic acids, in *A. thaliana*. 

## 2. Material and Methods

### 2.1. Source Plant Material, Extraction, Fractionation and Isolation of Pure Compounds

Rohitukine was extracted from dry leaf powder of *D. gotadhora*, as described previously by Mahajan et al. (2015) [15]. Briefly, shade-dried leaves were extracted thrice with 50% ethanol by sonication for one hour each at 45 °C, and the extract was dried using a rotary evaporator. The crude extract was then subjected to acid–base fractionation to obtain a fraction enriched with the alkaloid rohitukine and purified by repeated column chromatography over a silica gel mesh. The purity of the isolated rohitukine was confirmed by liquid chromatography–mass spectrometry (LC–MS) and high-performance liquid chromatography (HPLC). The purity of compounds was checked using HPLC by following the protocol of Kumar et al. (2016) [43]. An RP-C18 column was used (Neo Sphere, 250 mm × 4.6 mm, 5 μm). The mobile phase consisted of methanol–water (75:25 *v*/*v*) at flow rate of 1.0 mL/min. The temperature of the column oven was 40 °C and the injection volume was 4 μL. The detector used was a diode array detector, and the detection wavelength was 254 nm. Solutions of pure rohitukine were prepared by dissolving the alkaloid in sterile double-distilled water, followed by filter sterilization using a syringe filtration unit fitted with a 0.22 µm pore size (Millex-GV, Durapore). The filtered stock solution of rohitukine was then diluted appropriately in autoclaved double-distilled water for foliar treatment.

### 2.2. Plant Material and Growth Conditions of A. thaliana

Wild-type *A. thaliana* seeds (Col-0 background) were used in the experiment. The seeds were surface-sterilized with 70% ethanol, washed with autoclaved double-distilled water and sown on plates containing Murashige and Skoog (MS) basal medium (for media composition, see Table 1), sucrose 1.5% (*w*/*v*) and agar 0.6% (*w*/*v*) (Hi Media Laboratories Pvt. Ltd. Mumbai, India) [44], supplemented with appropriate rohitukine concentrations: 0 (control), 0.25 mM, 0.5 mM and 1.0 mM concentrations. For foliar treatment, plants were grown in plastic pots filled with 300 g autoclaved soil mixture (soil rite–sand–soil) at the ratio of (3:1:1). Seeds were kept at 4 °C in the dark for 48 h to ensure homogenous germination. After 48 h, the plates containing seeds were transferred to a growth chamber with the following conditions: photosynthetically active radiation (PAR): 680 μmole/m^2^/s; temperature: 24 °C; photoperiod light/dark cycles: 16/8 h; and relative humidity: 65%, in the Indian Institute of Integrative Medicine, Jammu and Kashmir, India. The plants grown in soil were irrigated with autoclaved distilled water at intervals of 24 h, while 1 mL of quarter-strength nutrient medium was applied at intervals of 48 h up to five weeks. 

### 2.3. Rohitukine Treatment for A. thaliana

Five-week-old plants of *A. thaliana* with uniform growth and maturity were exposed to 0.25 mM, 0.5 mM and 1.0 mM concentrations of pure rohitukine. Ten plants were treated with each concentration, and plants sprayed with autoclaved double-distilled water without rohitukine were used as controls. The plants were sprayed thrice with the appropriate concentrations of rohitukine dissolved in 50 mL autoclaved double-distilled water (1 mL to each plant) at intervals of 24 h, and 1 h post-treatment the samples were collected for analysis. Rohitukine is polar in nature, with a water solubility of >10 mg/mL and lipophilicity (log D) of <1.0 [10].It was found to have pH-dependent solubility, with its highest solubility in simulated gastric fluid (SGF) [45]. The reported value of acidity constant (pKa) is 5.83 [10]. We prepared the spray reagent at around pH 7.0 and it is therefore expected to be present at unionized form in solution, leading to higher absorption/permeation.

### 2.4. Determination of Rohitukine Content in A. thaliana

The content of rohitukine in treated samples was determined by thoroughly washing an equal quantity (200 mg) of rosette leaves, followed by homogenization in HPLC grade methanol and sonication, after which the homogenate was centrifuged at 12,000 rpm for 10 min at room temperature (RT). The supernatant was filtered through a syringe filter of 0.22 µm pore size and analysed following the protocol of Kumar et al. (2016) [43], using a HPLC system (Shimadzu, UFLC) consisting of a quaternary pump with a vacuum degasser, a thermos tatted column compartment, an autosampler and a PDA detector. A reverse-phase column (Lichrosphere RP C18e, 5 µm, 250 mm× 4 mm) was used and the column temperature was maintained at 40 °C. The HPLC mobile phase consisted of two solutions. Solution A was composed of water with 0.1% formic acid. The solution was filtered through a 0.45 µm membrane filter and degassed in a so nicator for 3 min. Solution B was pure HPLC-grade acetonitrile. The mobile phase was run using gradient elution: 0.01 min, 10% B; in the next 20 min, 50% B; in the next 5 min, 70% B; in the next 5 min, 90% B, and maintained at 80% B for 5 min; in the next 5 min, 10% B; followed by an equilibration period of 5 min. The flow rate was 0.8 mL/min, and the injection volume was 20 µL. The eluents were detected and analysed at 254 nm. A quantity of 1mg/mL of pure rohitukine was taken as standard.

### 2.5. Measurement of Leaf Area and Plant Weight

Leaf area was measured using the easy leaf area tool described by Easlon et al. (2014) [46]. Briefly, *A. thaliana* rosette leaves treated with rohitukine were photographed by placing them on plain paper along with a red-coloured piece of paper of 4 cm^2^, and measurements were made using easy leaf area software by following the instructions provided. The fresh weight of the aerial parts (rosette leaves) of plants was determined immediately after harvesting. For dry weight, the samples were completely dried in an oven until weight was constant, then weighed to obtain the dry weight using a fine weighing balance (Mettler Toledo, Columbus, OH, USA). 

### 2.6. Photosystem II Activity

Chlorophyll fluorescence parameters were assessed for the rohitukine-treated plants of *A. thaliana* using a Junior-PAM chlorophyll fluorometer (Heinz Walz, Effeltrich, Germany), following the manufacturer’s instructions. The parameters assessed were: actual photosystem II (PSII) efficiency (Φ PSII), intrinsic PSII efficiency (Fv/Fm), maximum PSII efficiency (Fv/Fm), photochemical quenching (qP), non-photochemical quenching (NPQ) and electron transport rate (ETR). ETR was calculated usingthe formula Φ PSII × photosynthetic photon flux density × 0.5 × 0.84, as given in [47].

### 2.7. Photosynthetic Pigment Quantification

Total chlorophyll content was determined in *A. thaliana* plants exposed to 0.25 mM, 0.5 mM and 1.0 mM rohitukine by adopting the method of Arnon (1949) [48], with minor modifications. Briefly, 200 mg fresh leaf samples were homogenized in 0.2 mL 80% acetone, followed by centrifugation at 12,000 rpm for 5 min. The supernatants were collected and their absorbances were recorded at 663 and 645 nm for chlorophyll content using a spectrophotometer.

### 2.8. Histochemical Detection of ROS 

The accumulation of H_2_O_2_ and O_2_^−^ was analysed by the histochemical staining method, as described by Shi et al. (2010) [49], using 3,3-diaminobenzidine (DAB) (Sigma-Aldrich, St. Louis, MO, USA, cat. no. D5637) and nitro blue tetrazolium (NBT) (Hi Media, cat. no. RM578), respectively. For H_2_O_2_ detection, a solution of 1mg/mL DAB was prepared in 10 mM phosphate buffer, and the pH was adjusted to 3.8 with1N HCL. *A. thaliana* leaves of similar maturity levels were immersed in DAB solution at RT in the dark until brown spots were visible. After six hours of staining, the samples were incubated in a mixture of ethanol, acetic acid and glycerol (3:1:1) at 80 °C for 15 min to bleach out chlorophyll for proper visualization. ForO_2_^−^ localization, the samples were immersed in a 1 mg/mL solution of NBT prepared in 10 mM phosphate buffer (pH 7.8) until blue-coloured spots appeared. The immersed samples were then boiled in ethanol for better visualization, and photographs were taken by placing the stained leaves in a clean place with a white background using a digital camera. 

### 2.9. The Activity of Antioxidant Enzymes

Fresh leaf tissue of rohitukine-treated and untreated (control) samples of *A. thaliana* were homogenized in potassium–phosphate extraction buffer (100 mM, pH 7.0), using a precooled pestle and mortar. The extraction buffer was composed of 0.05% (*v*/*v*) Triton X-100 and 1% (*w*/*v*) polyvinylpyrrolidone (PVP). The activity of ascorbate peroxidase (APX; EC, 1.11.1.11) was calculated following the protocol described by Asgher et al. (2014) [50]. Briefly, APX activity was determined by the decrease in absorbance of ascorbate at 290 nm due to its enzymatic breakdown. A total of 1 mL mixture of 50 mM phosphate buffer (pH 7.0), 0.5 mM ascorbate, 0.1 mM EDTA, 0.1 mM H_2_O_2_ and enzyme extract was prepared. The calculation of APX activity was carried out using the extinction coefficient 2.8 mM^−1^ cm^−1^, and one unit of enzyme was required to decompose 1 µmol of substrate per min at 25 °C.

### 2.10. RNA Extraction, cDNA Synthesis and qRT-PCR 

Rohitukine-treated leaves of *A. thaliana* were used as source material for RNA isolation, using TRizol reagent (Ambion, Life Technologies, Carlsbad, CA, USA), according to the manufacturer’s protocol. RNA quality and concentration were assessed on a 2% agarose gel and nanodrop spectrophotometer (Thermo Fisher Scientific, Waltham, MA, USA) by measuring the absorbance ratio at 260/280 nm. The isolated RNA samples were subjected to DNase (Ambion TURBO DNA-free, Life Technologies, Carlsbad, CA, USA) treatment to remove traces of genomic DNA. DNase-treated RNA samples were reverse-transcribed using a cDNA synthesis kit (Promega, Madison, WI, USA), following the manufacturer’s instructions, with oligo (dT) primers and 1μg of DNase-treated RNA used as a template. Primer pairs were designed from coding sequences (CDSs) of selected antioxidant system genes of *A. thaliana* (Table 2). The qRT-PCR reactions were set in the CFX96^TM^ Real-Time PCR Detection System (Bio-Rad, Hercules, CA, USA), based on SYBR green chemistry. The PCR reaction mixtures (10 μL) comprised SYBR Green Master mix at a volume of 5.0 μL, 1.0 µM of each primer (Integrated DNA Technologies, Coralville, IA, USA), appropriately diluted cDNA as template and MQ water was used to make up the final volume of 10 μL. Thermoprofiles of the reactions for qRT-PCR were included by preincubation at 95 °C for 10 min, followed by 45 cycles of 3-step amplification with melt (95 °C for 10 s, 60 °C for 10 s and 72 °C for 15 s) and melt. The normalization of the reaction was achieved using the primers of the actin gene as a control. The analysis of samples was carried out in triplicate, and the specificity of each primer pair was authenticated by a dissociation curve (a single peak was observed for each primer pair). The generated (threshold cycle) CT values were then transferred to Microsoft Excel and the 2^−ΔΔCT^ method of relative quantification was used to determine the quantitative variation between the samples examined [51].

### 2.11. Metabolite Extraction and Enrichment Analysis 

Metabolite quantification for *A. thaliana* samples (treated and control) was carried out using the method described by Lisec et al. (2006) [52], with minor changes. Briefly, 200 mg of fresh leaves from all four samples of *A. thaliana* treated with 0.25 mM, 0.5 mM and 1.0 mM rohitukine concentrations and the untreated control were excised using a fine pair of scissors and crushed in liquid nitrogen to a fine powder. Afterwards, 3 mL precooled HPLC-grade methanol (100%) was added and the homogenate was transferred to a glass vial. Then, 100 µL of ribitol (2 mg/10 mL) (Sigma-Aldrich, St. Louis, MI, USA) was added as an internal standard, followed by incubation at 70 °C for 10 min with continuous shaking. After incubation, the homogenate was centrifuged for 10 min at 15,000 rpm in the cold centrifuge; the supernatant was collected and 1.5 mL chloroform and 3 mL precooled autoclaved double-distilled water was added and slight vortexed. The upper polar and lower nonpolar layers were transferred to new well-labelled glass vials and dried over a rotary evaporator. The dried samples were then derivatized by adding 80 µL of 20 mg/mL Methoxy amine hydrochloride (Sigma-Aldrich USA), prepared in pyridine, to each tube, followed by incubation at 37 °C for 2 h. N, O-Bis (trimethylsilyl) trifluoroacetamide (BSTFA) (derivatization-grade) (Sigma-Aldrich USA) was added and another incubation at 37 °C took place for 1 h. After incubation, the mixture was transferred to suitable GC vials with inserts and analysed using GC–MS. 

The sample was analysed using a GC–MS 4000 system (Varian, Crawley, UK) equipped with a Supelco capillary column (15 m × 60.32 mm × 60.25 m). A 1 µL volume of derivatized sample was injected; the injection temperature was 230 °C, the interface was set to 150 °C and the ion source was adjusted to 250 °C. The program of gradient temperature was: initial temperature of 40 °C for 6 min, +10 °C/min up to 300 °C and hold at 300 °C for 6 min. Mass spectrometry was determined by the full-scan method, ranging from 35 to 780 (*m*/*z*). The metabolites were identified by comparison of mass spectra with the NIST17 library using the molecular ion masses (*m*/*z*) of the fragments and retention time indexes (RI). The downstream analysis of each metabolite was carried out with the peak area of each metabolite taken as source data, using the MetaboAnalyst 5.0 online tool [53].

## 3. Results

### 3.1. Qualitative and Quantitative Assessment of Isolated Rohitukine 

**Figure 1:**
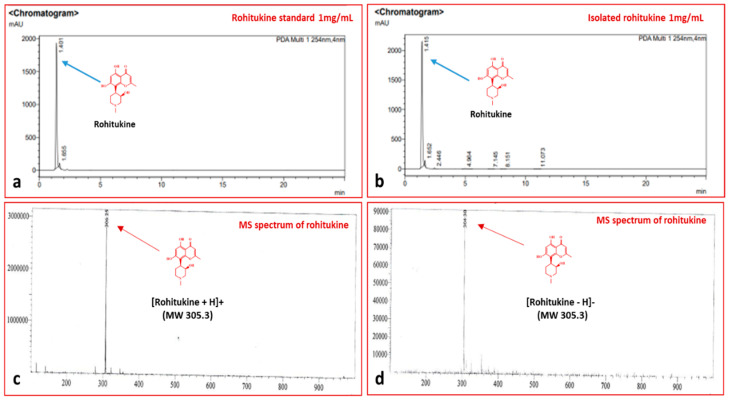
HPLC and MS chromatograms showing the purity of rohitukine isolated from leaves of *D. gotadhora.* (**a**) Chromatogram of standard rohitukine, 1 mg/mL. (**b**) Peak of rohitukine isolated from *D. gotadhora*. (**c**,**d**) Mass spectra showing the presence of a single peak of rohitukine (MW 305.3).

A quantity of 1.6 g of rohitukine was obtained after purification from 200 g shade-dried *D. gotadhora* leaves, i.e., 0.8% dry weight. After purification, the compound was subjected to a purity check by TLC, HPLC and LC–MS, in which the isolated compound was found to have 98% purity compared with the standard (Figure 1).

### 3.2. Determination of Rohitukine Uptake by A. thaliana

**Figure 2:**
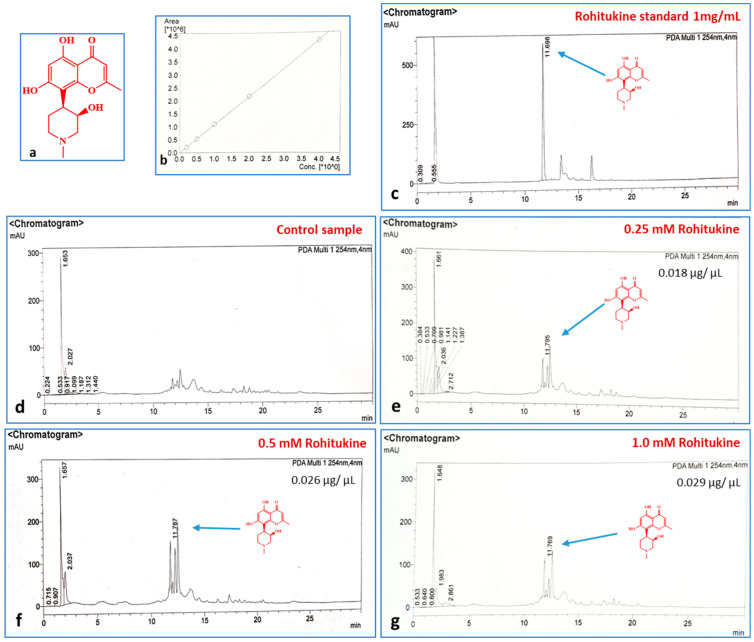
Detection and quantification of rohitukine in treated *A. thaliana* samples. (**a**) Structure of rohitukine. (**b**) Calibration curve of standard rohitukine. (**c**) Rohitukine standard, 1 mg/mL. (**d**) *A. thaliana* control peaks showing no traces of rohitukine. (**e**–**g**) Peaks indicating the presence of rohitukine at 0.25 mM, 0.5 mM and 1.0 mM concentrations.

After 24 h of treatment, the samples were subjected to HPLC for detection and quantification of rohitukine. In leaf tissues treated with 0.25 mM, 0.5 mM and 1.0 mM rohitukine, 0.009%, 0.013% and 0.014% of rohitukine was found to have accumulated, respectively (Figure 2), whereas in the control samples we did not find a peak corresponding to rohitukine, indicating its absence. A standard sample of 1mg/mL rohitukine was used for the calibration curve. However, in plants that received 1.0 mM of rohitukine treatment, the amount of assimilated rohitukine was of a higher quantity as compared to the lesser amount obtained with the 0.25 mM concentration. Therefore, the uptake and stability of rohitukine was confirmed.

### 3.3. Effect of Rohitukine on Plant Morphology

**Figure 3:**
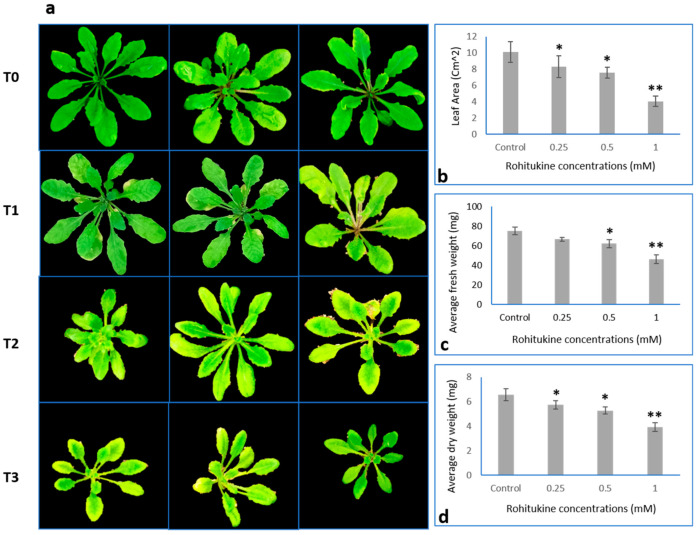
Morphology of *A. thaliana* plants treated with T0 (Control), T1 (0.25 mM), T2 (0.5 mM) and T3 (1.0 mM) rohitukine concentrations. (**a**) Five-week-old *A. thaliana* plants with and without rohitukine treatment. (**b**) Effects of 0.25 mM, 0.5mM and 1mM Rohitukine on leaf area of *A. thaliana* plants. (**c**) Average fresh weight of plants. (**d**) Effect of 0.25 mM, 0.5mM and 1mM Rohitukine on the dry weight of *A. thaliana.* The results are the means of more than three replicates expressed as means ± SD values. Statistical significance was determined by Student’s *t*-test. Asterisks * and ** denote the significance level of values at *p*-values < 0.5 and 0.05, respectively.

The *A. thaliana* plants treated with rohitukine showed concentration-dependent growth inhibition as compared to the control. A significant decrease in leaf area of 17.7%, 25.1%, and 60.3% with the0.25 mM, 0.5 mM and 1.0 mM rohitukine concentrations, respectively, was observed as compared to the untreated group. The fresh weight of plants treated with 0.25 mM, 0.5 mM and 1.0 mM of rohitukine decreased by 11.6%, 17.4% and 38.7%, respectively. Similarly, dry weight was found to be reduced by 12.5% 19.5% and 40.2% with 0.25 mM, 0.5 mM and 1.0 mM rohitukine, respectively (Figure 3). The maximum growth inhibitory effect of rohitukine was observed in *A. thaliana* plants treated with a 1.0 mM concentration. 

### 3.4. Influence of Rohitukine on Chlorophyll Content and PSII Activity 

**Figure 4:**
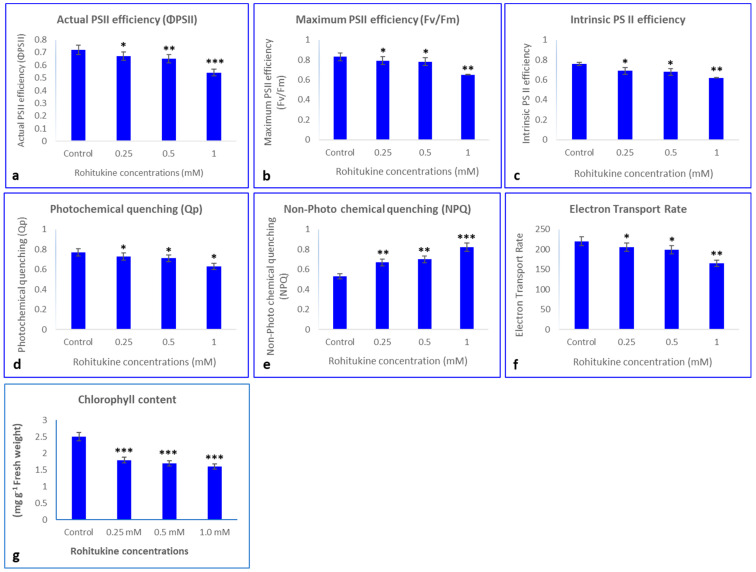
Effects of 0.25 mM, 0.5 mM and 1.0 mM rohitukine on (**a**) actual PSII efficiency, (**b**) maximum PSII efficiency, (**c**) intrinsic PSII efficiency, (**d**) photochemical quenching, (**e**) non-photo chemical quenching, (**f**) electron transport rate and (**g**) total chlorophyll content in five-week-old *A. thaliana* plants treated with rohitukine. Data are presented for the treatments as means ± SDs (*n* = 3). Statistical significance was determined using Dunnett’s multiple comparisons test. Asterisks *, ** and *** denote significance level at *p*-values < 0.5, 0.05 and 0.01, respectively.

Total chlorophyll content and PSII activity were studied in *A. thaliana* exposed to different concentrations of pure rohitukine by comparing it with untreated control plants. Plants treated with rohitukine showed a dose-dependent decrease in chlorophyll content. With 0.25 mM, 0.5 mM and 1.0 mM rohitukine concentrations, total chlorophyll content decreased by 1.8, 1.7 and 1.6 mg/g fresh weight (FW), respectively, as compared to the untreated control plants having 2.5 mg/gFW (Figure 4g). Actual PSII efficiency (Φ PSII), maximum PSII efficiency (Fv/Fm), intrinsic PSII efficiency, photochemical quenching (qP) and electron transport rate (ETR) decreased by 6.9%, 4.81%, 9.21% and 5.19%, respectively, under 0.25 mM rohitukine. On the other hand, non-photochemical quenching (NPQ) increased by 26.4% compared to the control. Application of 0.5 mM rohitukine decreased Φ PSII, Fv/Fm, Intrinsic PSII efficiency, qP and ETR by 9.7%, 6.02%, 10.52% and 7.79%, respectively, and non-photochemical quenching increased by 32% when compared to the control. Plants that received 1.0 mM rohitukine exhibited maximally decreased Φ PSII, Fv/Fm, Intrinsic PSII efficiency, qP and ETR, by 25%, 21.68%, 18.42% and 18.18%, respectively, and a 54% increase in NPQ as compared to the control (Figure 4).

### 3.5. Accumulation of O_2_^−^ and H_2_O_2_ in A. thaliana Plants in Response to Rohitukine

**Figure 5:**
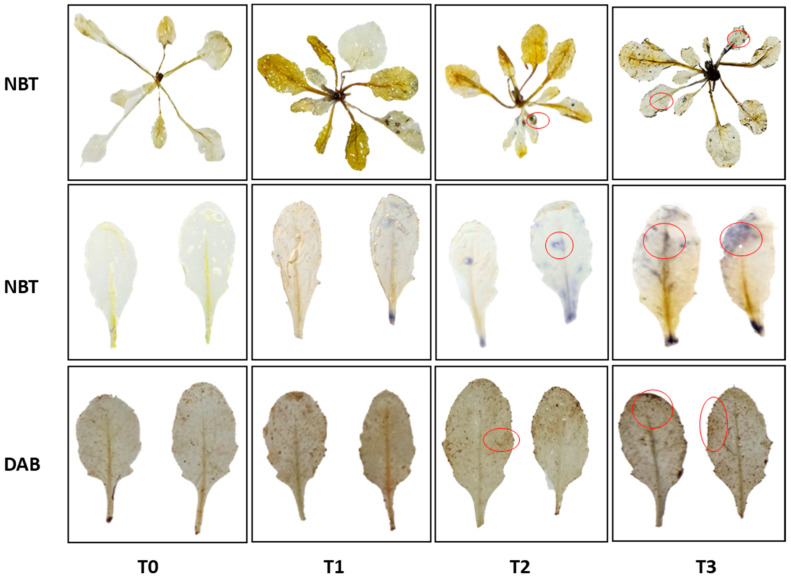
The effects of rohitukine concentrations on T0 (control), T1 (0.25 mM), T2 (0.5 mM) and T3 (1.0 mM) as detected through histochemical detection of ROS in *A. thaliana* leaves. Superoxide ions were detected with NBT staining dye; the H_2_O_2_ visualization in leaves was performed via DAB staining.

Increased levels of O_2_^−^ were noticed in *A. thaliana* plants as dark blue-coloured spots scattered on the leaves treated with rohitukine as compared to the untreated control. Similarly, the *A. thaliana* plants treated with pure rohitukine showed a significant increase in brown-coloured spots when immersed in DAB staining dye. These scattered brown spots were indications of increased levels of H_2_O_2_ in rohitukine-treated leaves as compared to untreated samples (Figure 5). Asin animal cells, rohitukine induces ROS in plant tissues, with maximum ROS observed for the1.0 mM treatment.

### 3.6. Effect of Rohitukine on Antioxidant Enzyme Activity

**Figure 6:**
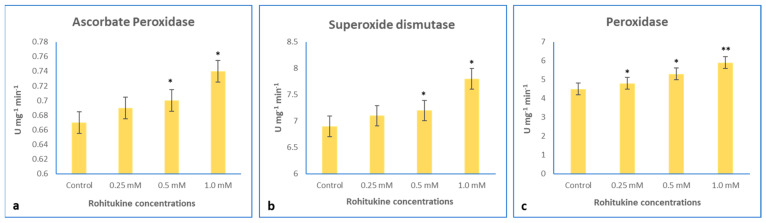
Effects of 0.25 mM, 0.5 mM and 1.0 mM rohitukine on (**a**) APX, (**b**) SOD and (**c**) POD of five-week-old *A. thaliana* plants treated with rohitukine. Data are presented for the treatments as means ± SDs (*n* = 3). Statistical significance was determined by Dunnett’s multiple comparisons test. Asterisks * and ** denote significance level at *p*-values < 0.5 and 0.05, respectively.

The activity of APX, SOD and POD increased by 2.9%, 2.8% and 6.6%, respectively, with the 0.25 mM rohitukine treatment, whereas with the0.5 mM treatment, the activity of APX, SOD and POD increased by 4.47%, 4.34%, and 17.7%, respectively. The application of the 1.0 mM rohitukine concentration maximally increased APX activity by 10.44%, SOD by 13.04% and POD by 31.1% as compared to the untreated control (Figure 6).

### 3.7. Effect of Rohitukine on the Expression of Key Genes Involved in the Antioxidant System

**Figure 7:**
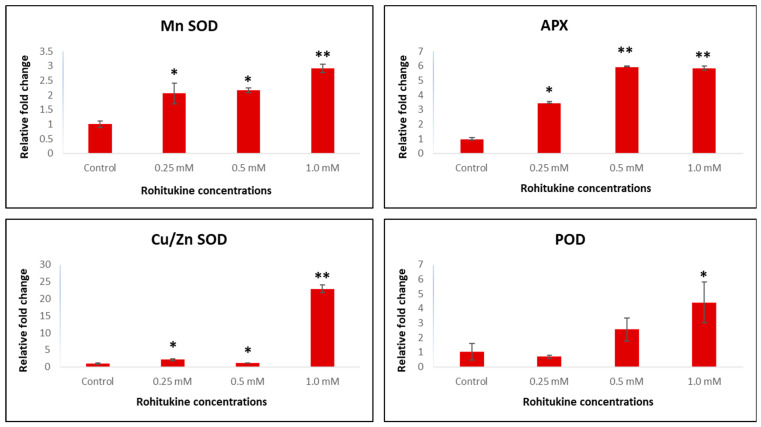
qRT-PCR-based expression analysis of key genes of the antioxidant system of *A. thaliana*. The bar diagrams represent the fold change expression of each gene upon 0.25 mM, 0.5 mM and 1 mM rohitukine exposure as compared to untreated controls. The results are presented as the means of three replicates and as means ± SDs. Statistical significance was determined by Student’s *t*-test. Asterisks * and ** denote the significance of fold changes at *p*-values < 0.05 and 0.005, respectively, as compared to untreated controls.

The expression patterns of key genes involved in the antioxidant system, such as manganese Mn-SOD, Cu/Zn-SOD, APX and POD, were analysed at transcript level in rohitukine-treated *A. thaliana* samples. The significantly increased expression of these genes was noticed in treated samples as compared to untreated controls. The Mn-SOD gene was found to have a two-fold increased expression in the 0.25 mM and 0.5 mM rohitukine treatments, while in the 1.0 mM treatment we noticed around a three-fold expression of Mn-SOD when compared to controls. Similarly, APX gene transcripts were found to have six-fold upregulated expression in the 0.5 mM and 1.0 mM treatments with rohitukine. In the treatment with the 0.25 mM concentration, the APX was found to be upregulated by 3.5-fold as compared to the control. In the treatment with the 1.0 mM rohitukine concentration, significantly higher transcripts of Cu/Zn-SOD were noticed in *A. thaliana* as compared to the control, whereas with the 0.25 and 0.5 mM rohitukine concentrations, the expression of Cu/Zn-SOD was found to be higher as compared to the control. The expression of the POD gene was also upregulated by rohitukine. The 0.5 mM and 1.0 mM concentrations of rohitukine increased the expression of POD by 2.5 and 4.5-fold, respectively (Figure 7). In all the treatments, overall expression profiles of Mn-SOD, APX, Cu/Zn-SOD and POD were increased when compared to controls. However, plants exposed to 1.0 mM of rohitukine showed the highest transcript levels for all the genes examined. 

### 3.8. Metabolite Profiling 

**Figure 8:**
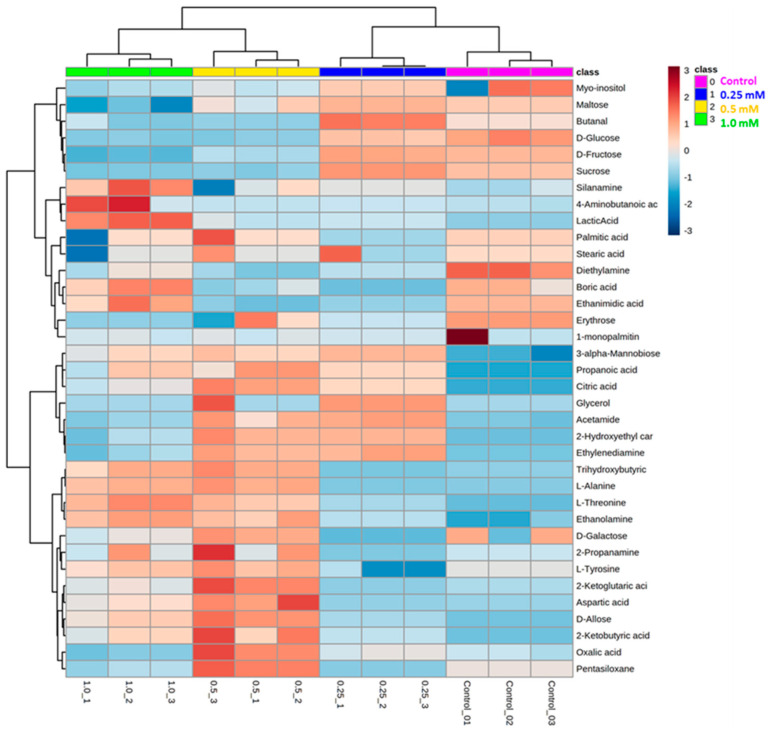
Heatmap illustration of quantities of commonly found metabolites in *A. thaliana* after 0.25 mM, 0.5 mM and 1.0 mM rohitukine treatments as compared to controls. The colour intensities of each box represent the level of each metabolite in each rohitukine-treated group.

With GC–MS analysis of rohitukine-treated *A. thaliana* leaf samples after derivatization and comparison of identified spectra with the NIST17 library, a total of 75, 73, 70 and 71 metabolites were identified with their known structures in the control, 0.25 mM, 0.5 mM and 1.0 mM rohitukine-treated samples, respectively. The metabolites identified were fatty acids, sugars, amino acids, organic acids, polyamines, carbohydrates, etc. To obtain the robust metabolome datasets, only those metabolites which were found to be present in at least three replicates were considered, and their presence in all four samples was ensured before processing. A total number of 37 metabolites were found to be common to all four samples (0.0 mM, 0.25 mM, 0.5 mM and 1.0 mM) and in each replicate. To understand the differences between samples and the similarity between replicates, and to determine the variables that contributed most to these differences, principal component analysis (PCA) was carried out for 37 metabolites, where PCA1 showed 41.8% and PCA2 showed 24.9% of the variation. The accumulation patterns of metabolites in the control as well as in all three treatments are shown by a heatmap generated from 37 metabolites present in all the samples (Figure 8). In all four major clusters, distinct patterns of altered levels of metabolite were reported. Therefore, the patterns of metabolite abundance and clustering indicate the metabolic changes caused by the exposure to rohitukine in *A. thaliana* leaves. 

Among sugars, the most frequent members found in all the four samples were: erythrose, fructose, galactose, glucose, maltose and sucrose. Dose-dependent decreased levels of fructose, glucose, maltose, sucrose and galactose were noticed in rohitukine-treated samples as compared to controls. Amino acids, L-alanine, aspartic acid, L-threonine and L-tyrosine were found with significantly higher concentrations in rohitukine-treated *A. thaliana* plants when compared to controls (Figure 8). Some other metabolites which were found to be significantly higher after rohitukine exposure were 3-alpha-Mannobiose, ethanolamine and silanamine. Meanwhile, the accumulation of several metabolites was found to be significantly lower after rohitukine exposure, including glycerol, Myo-inositol, acetamide, diethylamine, pentasiloxane, etc. Moreover, we also noticed changes in the levels of several organic acids in rohitukine-treated samples; the upregulated organic acids included: lactic acid, trihydroxy butyric acid and 4-amino butanoic acid. Conversely, several organic acid metabolites, such as palmitic acid, boric acid, stearic acid and oxalic acid, were found to have significantly lower concentrations in the 0.5 mM and 1.0 mM rohitukine treatments. 

## 4. Discussion

Although the biological activities of rohitukine in mammalian as well as in yeast strain cells have been deeply studied, the significance of this molecule in plant systems has not yet been elucidated. It is for the first time that we have tried to understand the physiological and biochemical impacts of rohitukine inside the *A. thaliana* model system. However, the rohitukine biosynthesis pathway in parent plants has not been elucidated yet. As far as the accumulation of rohitukine in source plant *D. gotadhora* is concerned, it was reported to accumulate in leaves, bark, fruits, seeds and twigs, but the highest concentration of rohitukine was reported in seeds (2.42%), followed by leaves (1.06%) [15,54]. Later, Kumar et al. (2016) [10] introduced the chromatography-free protocol of rohitukine isolation, in which they extracted 98% pure compound with a 1% (dry weight) yield. In the present study, we isolated 1.6 g of rohitukine from 200 g dry leaves of *D. gotadhora* with 98% purity (Figure 1). The pure compound of rohitukine was extracted from *D. gotadhora* (source plant) and applied to *A. thaliana*, where rohitukine interfered with plant growth and development. It is possible that rohitukine may leach out from the leaves of the parent plant during rain and it could also be found in soil samples as an allelochemical, which may also be due to the decomposition of plant tissues in soil. The leaching of toxic alkaloids from plant tissues into soil and drainage water has also been evidenced by previous studies [55,56].

For screening of the best inhibitory/modulatory concentration of rohitukine, we applied a range of rohitukine concentrations (0.01 mM to 10 mM) on *A. thaliana* seedlings. Among the concentrations screened so far, 1.0 mM rohitukine maximally inhibited the growth of *A. thaliana*, while 0.25 mM and 0.5 mM concentrations moderately affected growth as scored visually. Therefore, we applied 0.25 mM, 0.5 mM and 1.0 mM concentrations of rohitukine to analyse the dose-dependent effects of the molecule on *A. thaliana*. In previous studies, the dose-dependent growth inhibitory effects of extracts from an important medicinal plant *Hyptissuaveolens* were examined in several plant species [57].The stability and uptake of the compound were also assessed through HPLC, and rohitukine was found to be stable inside the plant tissues and uptake was also confirmed (Figure 3) [10,43]. The question related to the stability of rohitukine at various physiological pH levels is very important for its phytotoxic efficiency. In already published reports, the stability of rohitukine has been assessed by incubating the pure compound of rohitukine in buffers of different pH (1.2, 4.0, 6.8 and 7.4), bio-relevant fluids, such as SGF (pH 1.2) and simulated intestinal fluid (SIF) pH 6.8, and also in rat plasma. Rohitukine was found to be stable in all the tested conditions [10,43].

It has been reported that rohitukine is highly stable in diverse biological fluids. Moreover, it is found to be in the category of high-permeability molecules (log p_app_ > −5) based on Parallel Artificial Membrane Permeability Assay (PAMPA) data, which confirm passive transcellular permeation [52]. Computational analysis of rohitukine suggests that it is a substrate of P-gp, which is the key member of the ABC transporter system [45]. In addition, stress situations are reported to regulate transporter expression in the plant system [58]. The above-mentioned evidence in the literature indicates the possible uptake of rohitukine in biological systems. However, further investigations have to be performed to explore the exact mechanism of rohitukine uptake in *A. thaliana* [59]. Additionally, the plasma protein binding efficiency of rohitukine and its semisynthetic derivatives have also been reported [60]. However, in plants, the exact mechanism of membrane transport of rohitukine is unclear. An attempt has been made, using isolated epidermal cells, to understand the mechanism of alkaloid transport through plasma membranes, suggesting the involvement of transporter proteins [61].

The negative effects of plant secondary metabolites, such as L-mimosine, syringaldehyde, juglone, and vanillin, have already been assessed for *A. thaliana* and other crop species [62,63,64]. In the present study, rohitukine exposure significantly decreased total leaf area and plant biomass. Moreover, a moderate decrease in photosynthesis was observed with 0.25 and 0.5 mM rohitukine concentrations, while photosynthesis decreased maximally in plants that received 1.0 mM of rohitukine when compared to the controls. Similarly, a gradual decrease in total chlorophyll content was observed with increased rohitukine concentration. Similar results have been shown by Hussain and Reigosa (2021) [65], who investigated the influence of two plant secondary metabolites, ferulic acid and p-hydroxybenzoic acid, on the photosynthesis of *Rumex acetosa*, where both the molecules inhibited photosynthetic parameters, such as Fv/Fm, Φ PSII, qP and NPQ. Rutin is another secondary metabolite that is reported to inhibit Fv/Fm and the concentrations of chlorophyll pigments in *A. thaliana* [66]. At increased rohitukine concentrations, we noticed a decrease in ETR, Φ PSII, Fv/Fm, qP and intrinsic PSII efficiency in *A. thaliana*. Consequently, increased NPQ was observed upon rohitukine exposure. 

Generally, ROS are produced inside living cells when they encounter any external stress and activate the antioxidant defence system to overcome the such caused by oxidative stress [67]. In plants, ROS are present in •O_2_^−^ ionic states, such as hydroxyl radicals (•OH), and molecular states, including H_2_O_2_ and singlet oxygen (•O_2_) [68,69]. •O_2_^−^ is reported to increase during external stress and is the precursor of various ROS. The excessive generation of •O_2_^−^ causes an increment in ROS that leads to cell death [70,71]. Rohitukine exposure affected various physiological, biochemical and molecular mechanisms via the excessive production of ROS in *A. thaliana*. In rohitukine-treated *A. thaliana* plants, we detected the accumulation of •O_2_^−^ and H_2_O_2_ as blue- and dark brown-coloured spots, respectively, through the histochemical staining of leaves. The already published literature suggests that rohitukine induces ROS in yeast strains after 24 h of treatment [16]. Moreover, in cell lines, rohitukine and its semisynthetic derivatives have been reported to induce ROS-mediated apoptosis [21,72,73]. Similarly, two other alkaloids, Graveoline and vitrine, isolated from *Ruta graveolens* and *Evodilitoris*, with immunomodulatory, anti-inflammatory and anti-cancerous activities are reported to generate ROS in the root coleoptile of wheat [74,75]. We also noted the rohitukine-mediated induced expression of APX, SOD and POD, which are involved in the antioxidant defence system in plants. These genes are exclusively associated with ROS metabolism to combat the stress response. Therefore, it is likely that rohitukine may trigger cell death in plants via ROS generation and affect hormonal transport. Under all defined concentrations of rohitukine, there was a significant increment in APX, SOD and POD enzyme contents in *A. thaliana*. Khan et al. (2011) [76] investigated the SOD-, CAT- and POD-mediated growth inhibitory effects of aqueous and ethanol extracts of *Peganum multisectum* on ryegrass.

The reduction in maximum quantum yield of PSII shows that excitation energy trapping of PSII reaction centers was reduced. A decrease in photosynthesis was suggested to be due to stomatal closure [65]. Reduction in CO_2_ passage due to stomatal closure is responsible for the accumulation of ROS, the degradation of xanthophyll pigments and lipids and protein oxidation [77]. The slight decrease in PSII activity observed was due to more ROS accumulation, which increases oxygen production at 1 mM rohitukine. Moreover, the slightly higher ETRs recorded for treatments with 0.25 mM and 0.5 mM of rohitukine compared to the 1 mM treatment were due to less ROS accumulation, as revealed by DAB and NBT staining, which limits ROS production. Our results also show that rohitukine can block the electron acceptor to inhibit photosystem II. These results support the hypothesis that there was a reduction in photosystem II photochemistry and photosynthetic electron transport, which is responsible for ROS accumulation in *A. thaliana*. Similarly, secondary metabolites isolated from several endophytes inhibit PSII electron transport on the water-splitting enzyme and on the acceptor side between P680 and QA. The results of this study were confirmed by chlorophyll-a fluorescence measurements [78]. The reduction in chlorophyll fluorescence under metal stress shows HM antenna pigment disruption due to the hindrance of electron transport flow from PSII to PSI [79,80,81]. To address the phytotoxic effects of secondary metabolites, many studies have been published which show that secondary metabolites have impacts mainly through damage to photosynthetic machinery and frequent decomposition of photosynthetic pigments. Consequently, decrease in photosynthetic pigments leads to blockage of energy/electron transfer and inhibition of ATP synthesis [28].

The changes caused by any elicitor at the morphological and transcriptomic levels should be reflected in the final end products of gene and protein expression. The metabolome offers better visualization of the changes in the levels of many different metabolites. In recent years, metabolomics has gained considerable attention as a tool for acquiring better insight into the biological processes of organisms [82,83,84,85]. In an attempt to analyse the changes in metabolite levels caused by rohitukine in *A. thaliana*, GC–MS-based metabolomics was performed, in which amino acids, carbohydrates, other organic acids, etc., were identified. Among amino acids, L-alanine, aspartic acid, L-threonine and L-tyrosine were detected at higher concentrations in 0.5 mM and 1.0 mM rohitukine-treated samples. Generally, aromatic amino acids, such as tyrosine, play important roles in the synthesis of a wide range of secondary metabolites when plants encounter any external stress. L-tyrosine and other aromatic amino acids act as precursors for the synthesis of phenylpropanoids, a major group of plant secondary metabolites whose function is to protect the plant from abiotic stresses [86,87,88]. Many essential amino acids, such as valine, methionine, alanine, and leucine, as well as non-essential amino acids, such as histidine, proline cysteine, etc., have been reported to increase under abiotic and biotic stresses [89,90,91]. The metabolic analysis also identified dose-dependent decreases in concentrations of some sugars, including fructose, glucose, maltose, sucrose and galactose, in rohitukine-treated *A. thaliana* samples as compared to controls. The maximum decreased level of sugars was noticed with the1.0 mM rohitukine treatment. Disruption to photosynthesis and chlorophyll bleaching by rohitukine exposure in *A. thaliana* plants could be the reason for lesser concentrations of sugars in treated samples. There are several studies that have shown the negative impacts of extracts and pure compounds isolated from different plant species on recipient plants in the form of decreased levels of carbohydrates [92,93,94]. The concentration of myo-inositol was found to be slightly decreased upon rohitukine exposure. It has been reported that Myo-inositol participates in cellular functions and metabolism in plants [95]. It also mediates ROS-induced cell death in the presence of salicylic acid and ethylene towards stress tolerance [96]. Along with Myo-inositol, several other metabolites, such as acetamide, diethylamine, penta siloxane and glycerol, were found to have decreased levels. Indole-3-acetamide triggers stress responses in *A. thaliana* and participates in the crosstalk of auxin and abscisic acid [97]. 

## 5. Conclusions

In conclusion, we found that rohitukine not only perturbs physiological mechanisms in mammalian and yeast cells but also affects the growth parameters of *A. thaliana* by triggering ROS generation and metabolic changes and interfering with photosynthetic machinery. The inhibition of PSII activity combined with the upregulation of antioxidant system genes is most likely the basis of its property as an allelochemical. The activity of rohitukine inside *A. thaliana* tissues represents an example of a mechanism whereby active medicinal compounds exert their influence on multiple targets instead of single sites of action. The relative importance and the effectiveness of rohitukine perturbation inside the model plant *A. thaliana* give us an idea of how similarly plant and animal cells respond to the medicinally important molecule rohitukine. Moreover, the phytotoxic potential of rohitukine may help in maintaining the ecological interactions of the parent plant.

## Figures and Tables

**Table 1:** Composition of MS nutrient media used for the growth of *A. thaliana* plants.

Class/Category	Ingredients	mg/L
Macro elements	Ammonium nitrate	1650.000
	Calcium chloride	332.200
	Magnesium sulphate	180.690
	Potassium nitrate	1900.000
	Potassium phosphate monobasic	170.000
Microelements	Boric acid	6.200
	Cobalt chloride hexahydrate	0.025
	Copper sulphate pentahydrate	0.025
	EDTA disodium salt dihydrate	37.300
	Ferrous sulphate heptahydrate	27.800
	Manganese sulphate monohydrate	16.900
	Molybdic acid (sodium salt)	0.213
	Potassium Iodide	0.830
	Zinc sulphate heptahydrate	8.600
Vitamins	Myo-inositol	100.000
	Nicotinic acid (free acid)	0.500
	Pyridoxine HCl	0.500
	Thiamine hydrochloride	0.100
Amino Acid	Glycine	2.000

**Table 2:** Table showing the details of primers used for gene expression analysis.

S. No	Gene Name	Primer Sequence, Forward	Primer Sequence, Reverse	TM (°C)
1	Peroxidase (POD)	GAGTCAATCGAACAACAACATCC	CCTCTGTCTGAAACTCGTGC	60
2	Catalase (CAT)	TGGAAGAAGATGCAATTCGTGTT	CCAGGTCTTGGTCACATCG	60
3	Copper/zinc Superoxide dismutase (CU/Zn-SOD)	GAGATGATGGAACTGCCAC	TGGCTACTGGAAACGCAGG	60
4	Manganese Superoxide dismutase (Mn-SOD)	CAAGCTGTGAACAAGGGAGA	AGTGAGCGT CAATGG CACT	60
5	Ascorbate peroxidase (APX)	GCAGATGGGCTTATCTGAC	AGGCCTTCCTTCTCTCC	60
6	Actin 2	GTTGACTACGAGCAGGAG	CAGCAGCTTCCATTCCC	60

## Data Availability

Data are contained within the article.
